# Learning adaptive reaching and pushing skills using contact information

**DOI:** 10.3389/fnbot.2023.1271607

**Published:** 2023-09-14

**Authors:** Shuaijun Wang, Lining Sun, Fusheng Zha, Wei Guo, Pengfei Wang

**Affiliations:** State Key Laboratory of Robotics and System, Harbin Institute of Technology, Harbin, China

**Keywords:** reinforcement learning, pushing, contact information, adaptivity, task efficiency

## Abstract

In this paper, we propose a deep reinforcement learning-based framework that enables adaptive and continuous control of a robot to push unseen objects from random positions to the target position. Our approach takes into account contact information in the design of the reward function, resulting in improved success rates, generalization for unseen objects, and task efficiency compared to policies that do not consider contact information. Through reinforcement learning using only one object in simulation, we obtain a learned policy for manipulating a single object, which demonstrates good generalization when applied to the task of pushing unseen objects. Finally, we validate the effectiveness of our approach in real-world scenarios.

## 1. Introduction

Robotic Pushing is a fundamental manipulation skill in the field of robotics, finding utility in various scenarios such as scene rearrangement (Zeng et al., 2018), object manipulation, and environmental interaction. Efficient and adaptive object pushing skills are crucial for autonomous robotic systems to interact with their surroundings effectively (Bauza et al., 2018). To achieve successful object pushing, two main approaches have been explored in past research (Stüber et al., 2020): analytical-based methods and data-driven methods. While analytical methods rely on explicit knowledge of the forward and inverse kinematics and dynamics models of manipulation, data-driven approaches leverage machine learning techniques to acquire pushing skills through experience.

Analytical-based methods (Yoshikawa and Kurisu, 1991; Howe and Cutkosky, 1996; Dogar and Srinivasa, 2010; Zhu et al., 2017) require a clear understanding of the object's physical properties, including mass, center of mass, friction coefficients, and other contact parameters. Zhou et al. (2019) demonstrates the differentially flat nature of quasi-static pushing with sticking contact and an ellipsoid approximation of the limit surface. The pusher-slider system is effectively reducible to the Dubins car problem with bounded curvature, enabling easy trajectory planning and time-optimality. The paper proposes closed-loop control with dynamic feedback linearization or open-loop control using mechanical feedback from two contact points for trajectory stabilization. Lynch (1993)proposes a method for a robot to plan object manipulation through pushing. The approach involves estimating the geometry and friction properties of the object by conducting experimental pushes and observing the resulting motion. Additionally, the paper explores object recognition based on their distinctive friction parameters. The focus is on developing a practical framework for the robot to understand and interact with objects in its environment by learning their friction-related characteristics. Lynch and Mason (1996) addresses the problem of planning stable pushing paths for robots to position and orient objects in the plane, especially when grasping and lifting are not feasible due to the objects' size or weight. The study focuses on stable pushing directions and the controllability issues arising from non-holonomic velocity constraints, presenting a planner for finding such paths amidst obstacles, with practical demonstrations on various manipulation tasks. In Lloyd and Lepora (2021) researchers propose a reactive and adaptive method for robotic pushing using high-resolution optical tactile feedback instead of analytical or data-driven models. The method demonstrates accurate and robust performance on planar surfaces, and highlights the need to explore explicit models and test on non-planar surfaces for improved generalization. By explicitly modeling these characteristics, analytical methods can achieve precise control and manipulation of specific objects (Hogan et al., 2018). However, these methods often lack the adaptability to handle novel objects whose properties are not known a priori. Consequently, they struggle to generalize their pushing skills to diverse and previously unseen objects.

On the other hand, data-driven approaches (Bauza and Rodriguez, 2017; Eitel et al., 2020; Song and Boularias, 2020; Yu et al., 2023) have achieved significant attention in recent years, primarily due to their ability to adapt to different objects without prior knowledge of their physical properties. These approaches utilize machine learning algorithms, such as supervised learning (Li et al., 2018) and reinforcement learning (Raffin et al., 2021; Cong et al., 2022), to acquire pushing skills from experience. Supervised learning techniques can estimate unknown object properties (Mavrakis et al., 2020), such as the center of mass, while reinforcement learning enables the learning of continuous control policies for pushing actions (Xu et al., 2021). A novel neural network architecture for learning accurate pushing dynamics models in tabletop object manipulation tasks is proposed in Kim et al. (2023). The proposed model possesses the desirable SE(2)-equivariance property, ensuring that the predicted object motions remain invariant under planar rigid-body transformations of object poses and pushing actions. Extensive empirical validations demonstrate that this new approach outperforms existing data-driven methods, leading to significantly improved learning performances. In Kumar et al. (2023), Inverse Reinforcement Learning (IRL) is explored to learn the reward instead of designing it by humans, achieving better performance in the pushing tasks. Chai et al. (2022) introduces a novel large planar pushing dataset encompassing various simulated objects and a new representation for pushing primitives, facilitating data-driven prediction models. Additionally, it proposes an efficient planning method with a heuristic approach to minimize sliding contact between the pusher and the object, addressing challenges in reasoning due to complex contact conditions and unknown object properties. Paus et al. (2020) proposes an approach for parameterizing pushing actions in robotic tasks using internal prediction models. The method involves representing scenes as object-centric graphs, training the internal model on synthetic data, and evaluating it on real robot data to achieve high prediction accuracy and generalization to scenes with varying numbers of objects. Data-driven approaches have shown promising results in handling a wide range of objects, exhibiting better generalization and adaptability compared to analytical methods.

**Figure 1:**
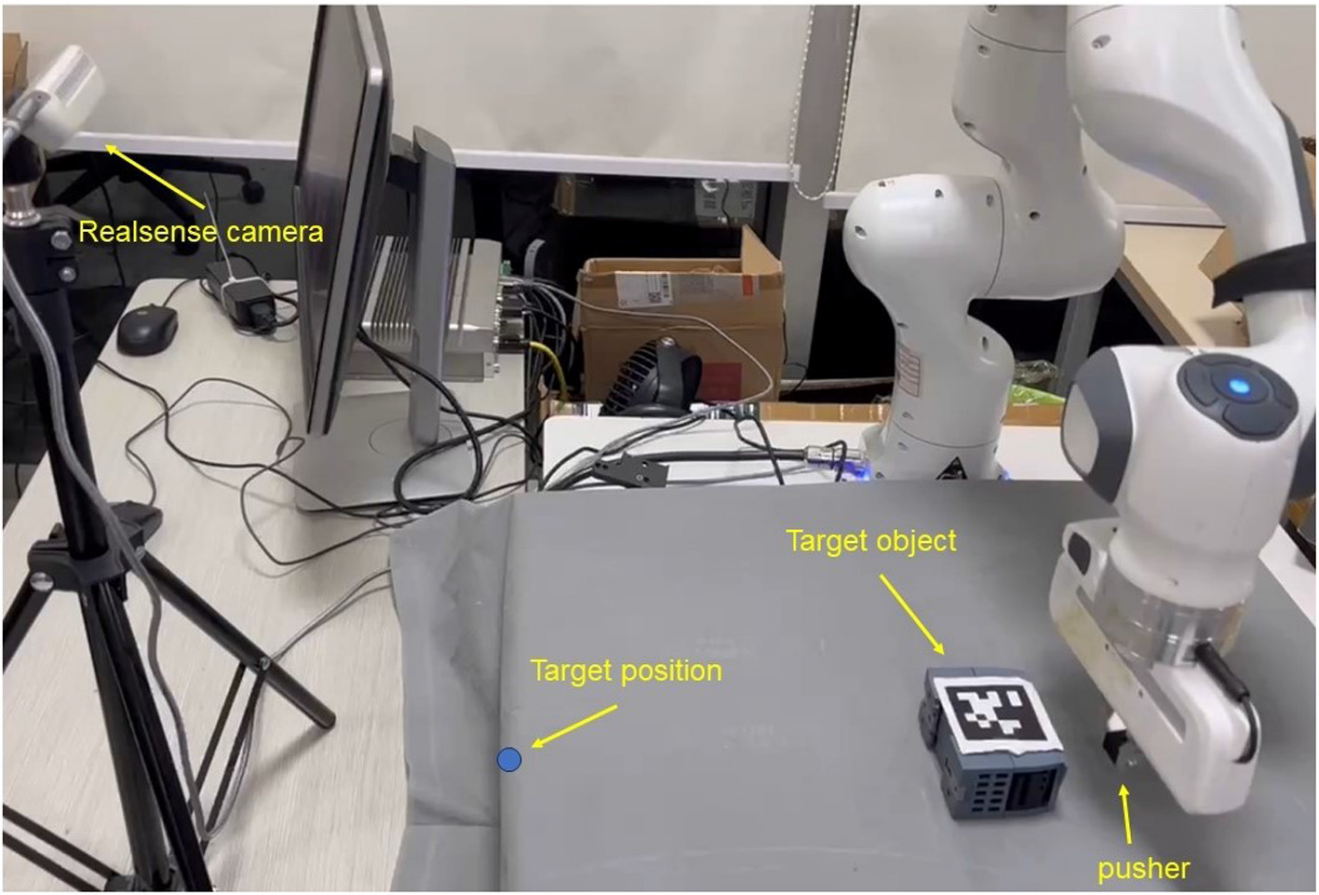
The robot pushing system: the object pose is detected by Apriltag techniques, and the pusher is the end effector of the franka robot. The objective of the task is for the pusher to move the object to the target position through pushing.

Reinforcement learning (RL) methods have recently gained popularity in low-level robot control tasks (Singh et al., 2022; Elguea-Aguinaco et al., 2023), as they enable the learning of task controllers using low-dimensional data as inputs, which are often easier to learn policies from compared to high-dimensional data, such as images. These pieces of information can be incorporated into RL as observations and can also be utilized in the design of RL reward functions. In this paper, we present a novel approach for object pushing based on deep reinforcement learning. Our method aims to efficiently push unknown objects from random initial poses to predetermined target positions using the contact information in the design of the reward function. The robot experiment system is shown in Figure 1.

We leverage contact force information between the pusher and the object to design an effective reward function that guides the learning process. Contact force information plays a critical role in pushing tasks, as it provides valuable cues about the interaction dynamics and the effectiveness of the applied forces. Surprisingly, contact force information has been underutilized in previous reinforcement learning-based pushing tasks (Dengler et al., 2022), especially in the reward design phase.

To address this limitation, we introduce appropriate observations and continuous action outputs that enable the agent to perform continuous and adaptive pushing control (Shahid et al., 2020). Our reward function design encourages the alignment between the direction of the contact force and the line connecting the object and the target. Furthermore, we incentivize minimizing the distance between the object's center of mass and the contact point where the force is applied. By doing so, we effectively encourage pushing actions that generate zero rotational torque, resulting in pure translation.

In our evaluation, we conducted extensive experiments in a simulated environment. Remarkably, our approach achieved higher task success rates, improved task completion efficiency, and demonstrated superior generalization capabilities compared to previous methods that did not consider contact force information. Notably, our training process utilized only one object, yet the learned policy successfully generalized to other unseen objects without prior knowledge of their physical characteristics. These findings demonstrate the effectiveness and potential of our method for real-world applications involving diverse objects and environments.

By leveraging contact force information and employing deep reinforcement learning, our proposed method contributes to improving adaptive reaching and pushing skills in robotics. The integration of contact force information in the reward function design provides valuable insights into the physical interactions involved in object pushing, leading to more precision and adaptive pushing policy.

In summary, our work highlights the importance of contact force information in object pushing tasks and presents a novel approach that effectively utilizes this information in the reward design. By combining deep reinforcement learning (DRL) and contact force guidance, we achieve improved task success rates, task completion efficiency, and generalization capabilities. Our research contributes to the ongoing efforts in enhancing robotic manipulation skills and lays the foundation for future advancements in autonomous systems interacting with diverse objects and environments.

The contributions of this paper are listed as follows:

- We introduce a RL-based reaching and pushing framework that uses only one object during training, allowing the trained policy to adapt effectively to unseen objects;
- We consider contact information in the design of the reward function to enhance task performance;
- We demonstrate that the learned policy, developed with our proposed approach, achieves higher success rates, task efficiency, and adaptability compared to policies that do not consider contact information.

## 2. Preliminaries

We model the object pushing task as a Markov decision process (MDP), which is defined by the tuple (S,A,T,R,γ), where:

S is the set of states, A is the set of actions, T is the transition function that specifies the probability of moving to a new state *s*′ given the current state *s* and action *a* taken, i.e.,


(1) $$\begin{array}{l} T\left(s,a,s^{\prime }\right)=\mathrm{Pr}\left(S_{t+1}=s^{\prime }|S_{t}=s,A_{t}=a\right) \end{array}$$

R is the reward function that maps a state-action pair (*s, a*) to a scalar reward *r*∈ℝ, and γ is the discount factor that determines the importance of future rewards. The goal of our learning system is to find a policy π:S→A that maximizes the expected cumulative discounted reward:


(2) $$\begin{array}{l} \overset{\infty }{\underset{t=0}{\sum }}\gamma^{t}R_{t+1} \end{array}$$

where *R*_*t*+1_ is the reward obtained at time *t*+1, and γ^*t*^ is the discount factor applied to future rewards at time *t*.

In this work, the SAC reinforcement learning method is applied. Soft Actor-Critic (SAC) Haarnoja et al. (2018) is a reinforcement learning algorithm that can be used to solve Markov decision processes (MDPs), which are mathematical models for decision-making problems. The goal of an agent in an MDP is to maximize its expected cumulative reward by taking actions in an environment.

SAC is based on the actor-critic architecture, which consists of an actor network that selects actions and a critic network that estimates the value of a state or state-action pair. However, SAC introduces several improvements over the traditional actor-critic algorithm, such as the use of a soft value function and entropy regularization.

The soft value function is defined as the expected sum of rewards plus a temperature-scaled entropy term. The entropy term encourages exploration and prevents premature convergence by penalizing deterministic policies. The temperature parameter can be learned during training or set manually.

The entropy regularization term is added to the actor objective to encourage exploration and to prevent the policy from becoming too confident. The overall objective of SAC can be written as:


(3) $$\begin{array}{l} L\left(\theta \right)=E_{\tau \sim \pi_{\theta }}\left[\overset{T}{\underset{t=0}{\sum }}r_{t}+\alpha H\left(\pi_{\theta }\left(\cdot |s_{t}\right)\right)\right]-E_{s_{t}\sim D}\left[V_{\phi }\left(s_{t}\right)\right] \end{array}$$

where θ and ϕ are the parameters of the actor and critic networks, respectively, τ is a trajectory sampled from the policy π_θ_, *r*_*t*_ is the reward at time step *t*, α is the temperature parameter, H is the entropy function, and D is the replay buffer containing past experiences.

The first term in the objective is the expected cumulative reward with entropy regularization, and the second term is the value function approximation error. The parameters are updated using gradient descent on the objective.

## 3. Methods

### 3.1. Proposed method framework

**Figure 2:**
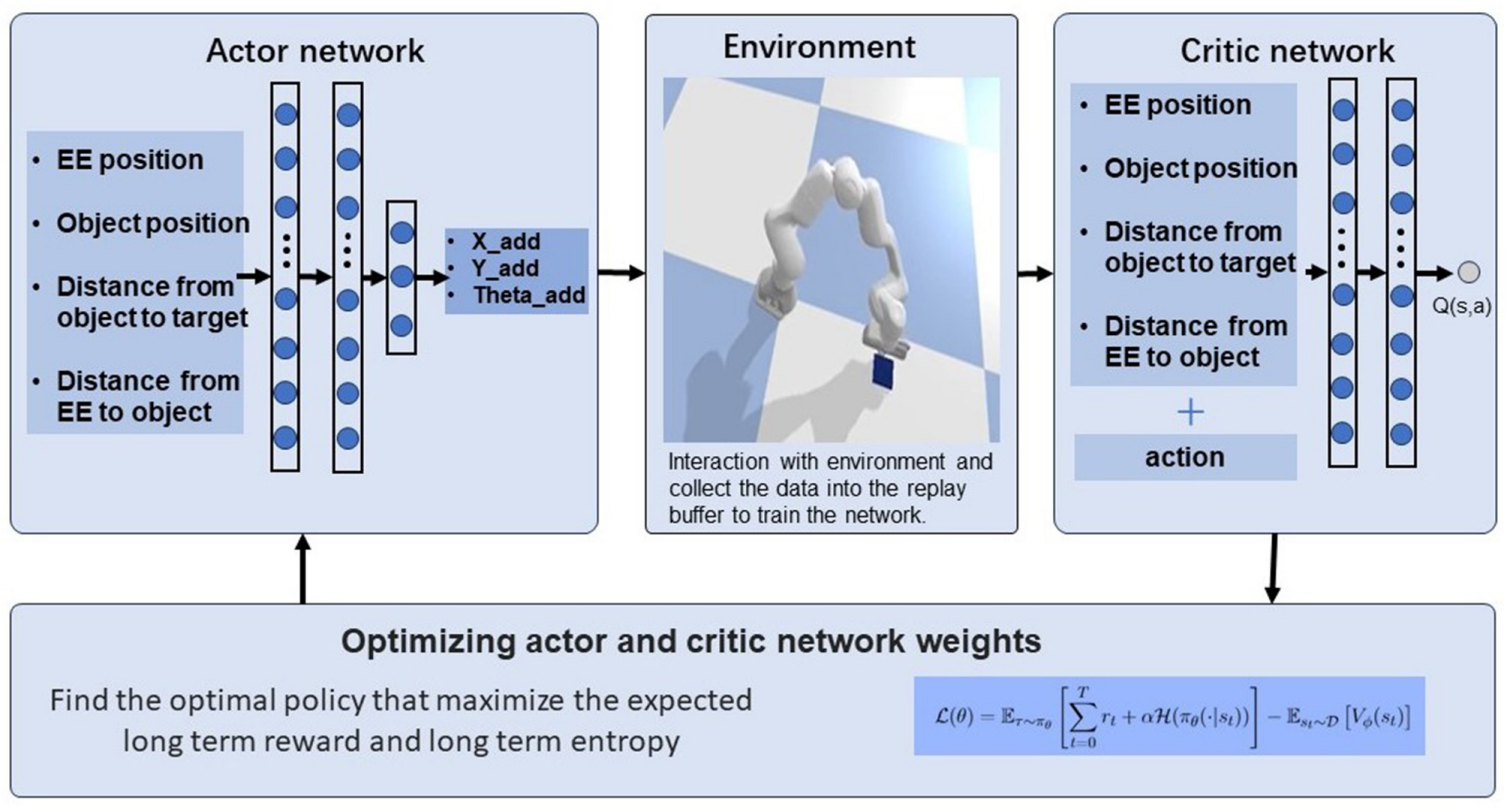
Framework of deep reinforcement learning for adaptive pushing using contact information.

In this work, we propose a manipulation framework namely “pusher”, which is an RL-based method to achieve objects reaching and pushing manipulation skills. The framework can be seen in Figure 2.

Our approach involves training a deep neural network policy using the Soft Actor-Critic algorithm (SAC) to learn continous pushing behaviors from sensory input. Specifically, we consider the contact information in the procedures of interaction with the environment in the reward design part.

Our framework involves three parts: actor-network, robot simulation environment and critic-network. In the training phase, we use a physics simulator to generate a dataset of pushing movements for a single object. We then use data collected in the progress to train a neural network policy (actor-network). During training, the policy learns to map raw sensory input to pushing actions that achieve the desired goal. The actor-network and critic network parameters are updated with the interaction with the environment.

**Figure 3:**
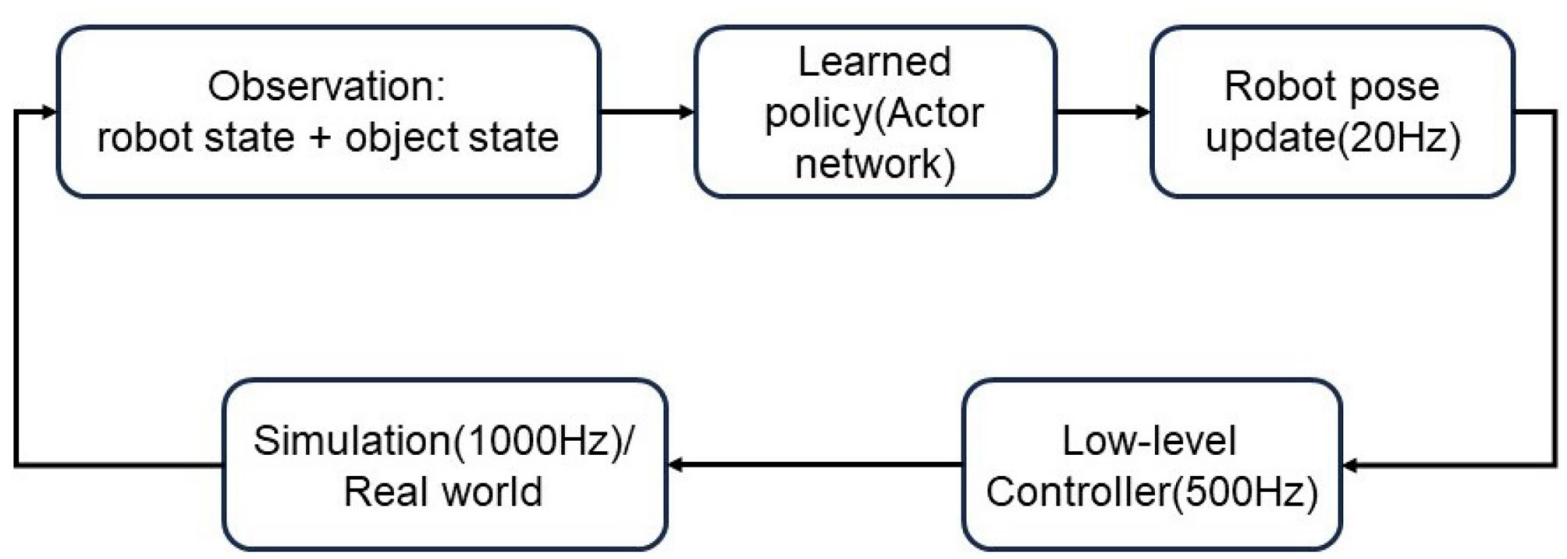
The control framework in the simulation, real-world, and joint-level control.

The control framework can be seen in Figure 3. The robot state and object state includes the end-effector position, object position, distance from object to target, and distance from end-effector to object. They are treated as input of the learned policy which is represented by the actor-network. The output of the policy is the action of the SAC algorithm. Robot pose is updated in frequency of 20 Hz. Inverse kinematics is applied to update the joint positions which are sent to the real robot in the real world or simulation.

### 3.2. State and action space

The observation space includes the position of the target object, the position and orientation of the end effector of the robot arm, as well as the distance between the target object and the end effector, and the distance between the target object and the target location. The action space is defined by the increments in the position and orientation of the end effector. Specifically, in this paper it is constrained to the planar space, specifically in the *x* and *y* directions, as well as the angle of rotation around the *z*-axis.

The observation space and action space of pushing can be formulated as follows:


(4) $$\begin{array}{l} Opush=\left[d_{1},d_{2},\theta ,p_{x},p_{y},e_{x},e_{y}\right] \end{array}$$

where *d*_1_ represents the distance of target object and end effector of the manipulator, *d*_2_ represents the distance of target object and target position. θ is rotation degree in *z*-aisx, *p*_*x*_, *p*_*y*_ is the position of target object, and *e*_*x*_, *e*_*y*_ is the position of the end effector of the manipulator.

The action space of pushing can be formulated as follows, which is an incremental action:


(5) $$\begin{array}{l} A=\left[\Delta x,\Delta y,\Delta \theta \right] \end{array}$$

where Δ*x*, Δ*y* represents the position increment of the end effector, and Δθ represents the orientation increment of the end effector. Once we obtain the incremental output from our policy, we add them to current robot position and rotation to update the control signals of the end effector in cartesian space.

### 3.3. Reward design

In reinforcement learning-based frameworks, the reward function plays a crucial role as it guides the learning policy toward desired states. In the context of manipulation tasks in this paper, which involves extensive interaction with the environment, considering contact information in the reward function becomes essential. Surprisingly, this aspect has received limited attention in previous reinforcement learning-based approaches.

**Figure 4:**
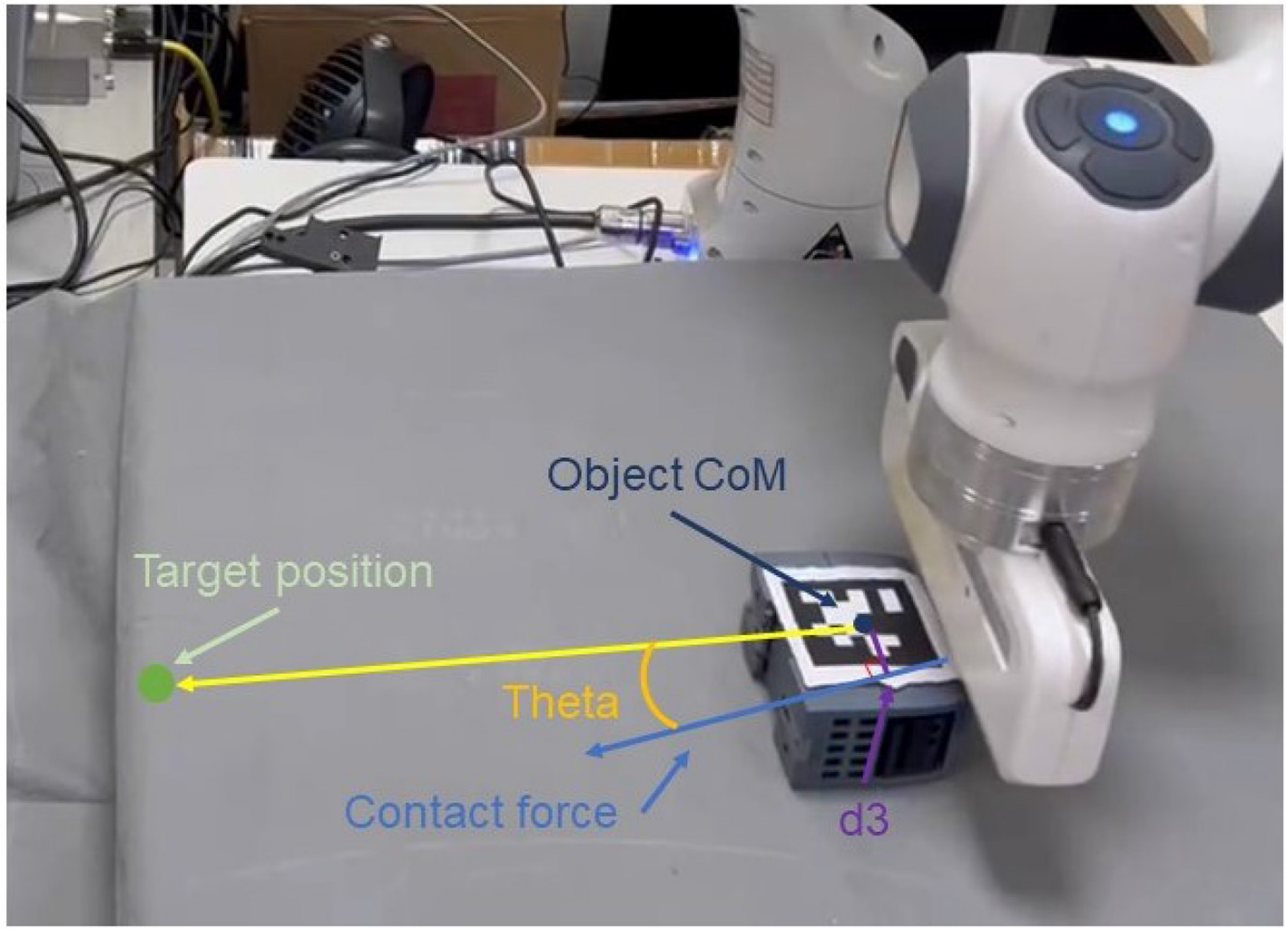
Contact model of interaction between object and pusher.

Firstly, let us introduce the contact model employed in our work. The contact model for pushing is represented in a straightforward manner, as depicted in Figure 4. It comprises the Center of Mass (CoM) of the target objects, their radius, and the orientation of the contact force. Notably, in this paper, we do not explicitly consider the magnitude of the contact force; Only the force direction is utilized.

In contrast to merely observing the state of the center of mass before and after the robot's interaction, incorporating contact geometric information at the time of contact can provide more precise and valuable insights. These geometric parameters offer advantages beyond determining the proximity of the target object to the desired position. They also contribute to a more accurate prediction of the potential closeness or distance of the target object to the desired state.

The mathematical representation of the reward functions for pushing in our work can be expressed as:


(6) $$\text{Reward}=\{\begin{array}{ll} -\theta -\lambda \times d3, & \text{if contact}\\ e^{-d3}-2, & \text{otherwise} \end{array}$$

In the formula, θ represents the angle between the contact force direction and the line connecting the object's center of mass to the target position. *d*3 refers to the distance from the object's center of mass to the contact force direction, which can be considered as the lever arm. λ is set to 30 in this work. As evident from this design, when there is no contact, we encourage the pusher and the object to make contact. Conversely, when there is contact, we incentivize the contact force to act through the object's center of mass, aligned with the line connecting the object to the target position, effectively encouraging the generation of zero rotational torque. This preference leads to the most direct path to push the object toward the target point.

### 3.4. Domain randomization and training procedures

In our study, we employed domain randomization to enhance the generalization of the trained reinforcement learning (RL) models. Specifically, we randomized the object's mass, friction coefficient, and initial position and orientation.

The reinforcement learning training process was conducted using the Soft Actor-Critic (SAC) algorithm. The hyperparameters used in the training were set as follows: a batch size of 100, a learning rate of 0.001, replay buffer is 1e6, discount factor is 0.99, interpolation factor in polyak averaging for target networks is 0.995, entropy regularization coefficient is 0.2. The PyBullet physics engine (Coumans and Bai, 2016) as the simulator, PyTorch as the learning framework, and Python as the programming language.

The Table 1 shows the range of values used for domain randomization.

**Table 1:** Domain randomization parameters for pushing tasks.

**Task**	**Parameter**	**Range**
Pushing	Object mass	(0.2, 0.5) kg
	Object friction	(0.5, 1.0)
	Object position	[(0.1, 0.2), (0.45, 0.55)] m
	Object orientation	(−0.15, 0.15) rad

We adopt the soft actor-critic (SAC) algorithm to learn the pushing tasks under the domain randomization framework. The training process involves the following steps as shown in the pseudocode implementation of the training procedure in Algorithm 1.

**Algorithm 1:**
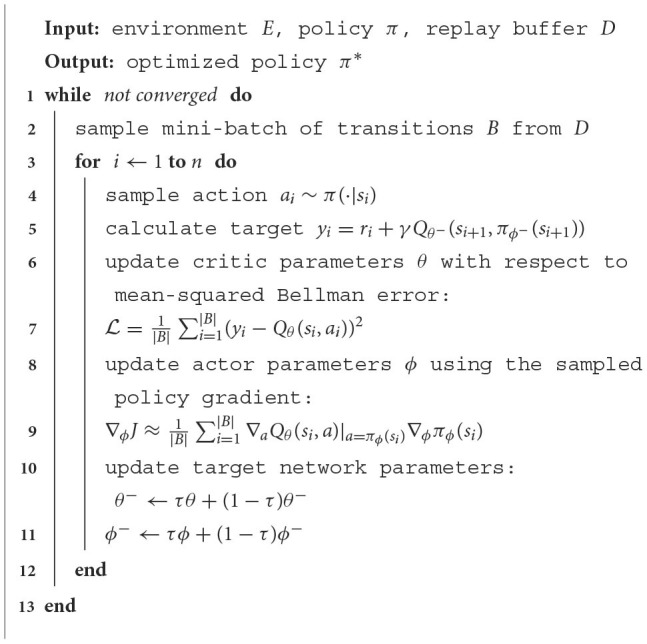
Soft actor-critic (SAC) training algorithm.

## 4. Experimental results and analysis

### 4.1. Experiments setup

The simulation and real experiments are conducted in this part to demonstrate the proposed method's performance. For simulation, we randomized several parameters of the objects to create a diverse training environment. Specifically, we randomized the mass, friction coefficient, and initial position of the objects. The mass and friction coefficient were randomly sampled from predefined ranges, and the initial position of the objects was randomly set within a specified region. This randomization process helps to introduce variability in the objects' properties, making the training environment more challenging and realistic. The use of randomized parameters allows the policy to learn to adapt to different object configurations, which can improve the policy's generalization performance.

**Figure 5:**
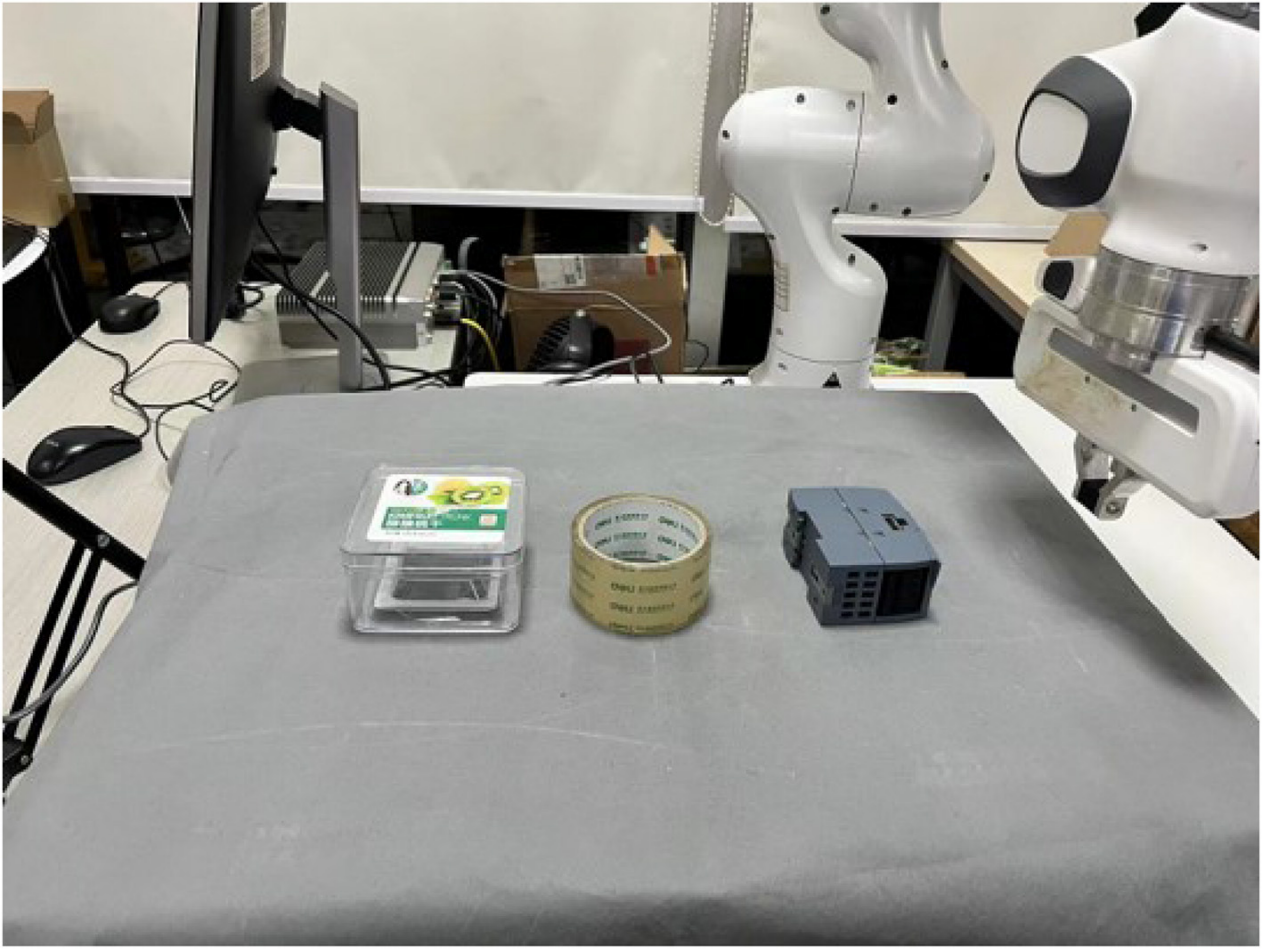
Real-world experiments setup and test objects with different mass, shape, and coefficient of friction.

As can be seen in Figure 1, in real experiments, an external camera is applied to obtain the pose of the target object. This is completed by the hand-eye calibration method (Tsai et al., 1989), transferring the target object pose in the camera coordination to the world coordinate. We use the Apriltag (Olson, 2011) attached to the target object to detect the pose of objects. For real experiments, we selected three representative objects to illustrate the effectiveness of our approach, as depicted in Figure 5.

### 4.2. Baseline

To highlight our proposed contact-aware based method, we compare it with a previous state-based method, which serves as the baseline approach. The state-based method and our proposed contact-aware-based method share the same observation but have different reward design manners. In the state-based method, it does not consider any contact interaction information in the reward design, in other words, the baseline method only takes state changement before and after the action is executed. Specifically, the baseline reward is designed as Equation (7):


(7) $$\text{Reward}=\{\begin{array}{ll} 1, & \text{if }d_{\text{current}}-d_{\text{last}}<0\\ e^{-d3}-2, & \text{otherwise} \end{array}$$

In Equation (7), *d*_*current*_ and *d*_*last*_ refer to the distance before and after action execution. *d*3 refers to the distance from the object's center of mass to the contact force direction as the same in Equation (6).

### 4.3. Performance analysis and discussion

#### 4.3.1. Success rate

We conducted experiments in the Pybullet simulation to showcase the performance of our proposed contact-aware method in achieving a higher task success rate by considering the contact information during interactions with the environment. We trained the model for 150 epochs, with 20,000 steps per epoch for both tasks. The environment is terminated once the task goal is satisfied, the object is out of the workspace, or the maximum number of steps per episode is reached. To evaluate the performance of our method, we used the task success rate, which is calculated by evaluating the learned policy 50 times at the end of each epoch.

In this section, we employed the task success rate as one of the evaluation metrics. The task success rate is defined as the percentage of episodes in which the robot successfully completed the task, based on the predefined criteria: a successful completion is recorded when the object is positioned within 2 cm of the target location. By measuring the task success rate, we were able to assess the effectiveness of our contact-aware method in improving the performance of the pushing task.

**Figure 6:**
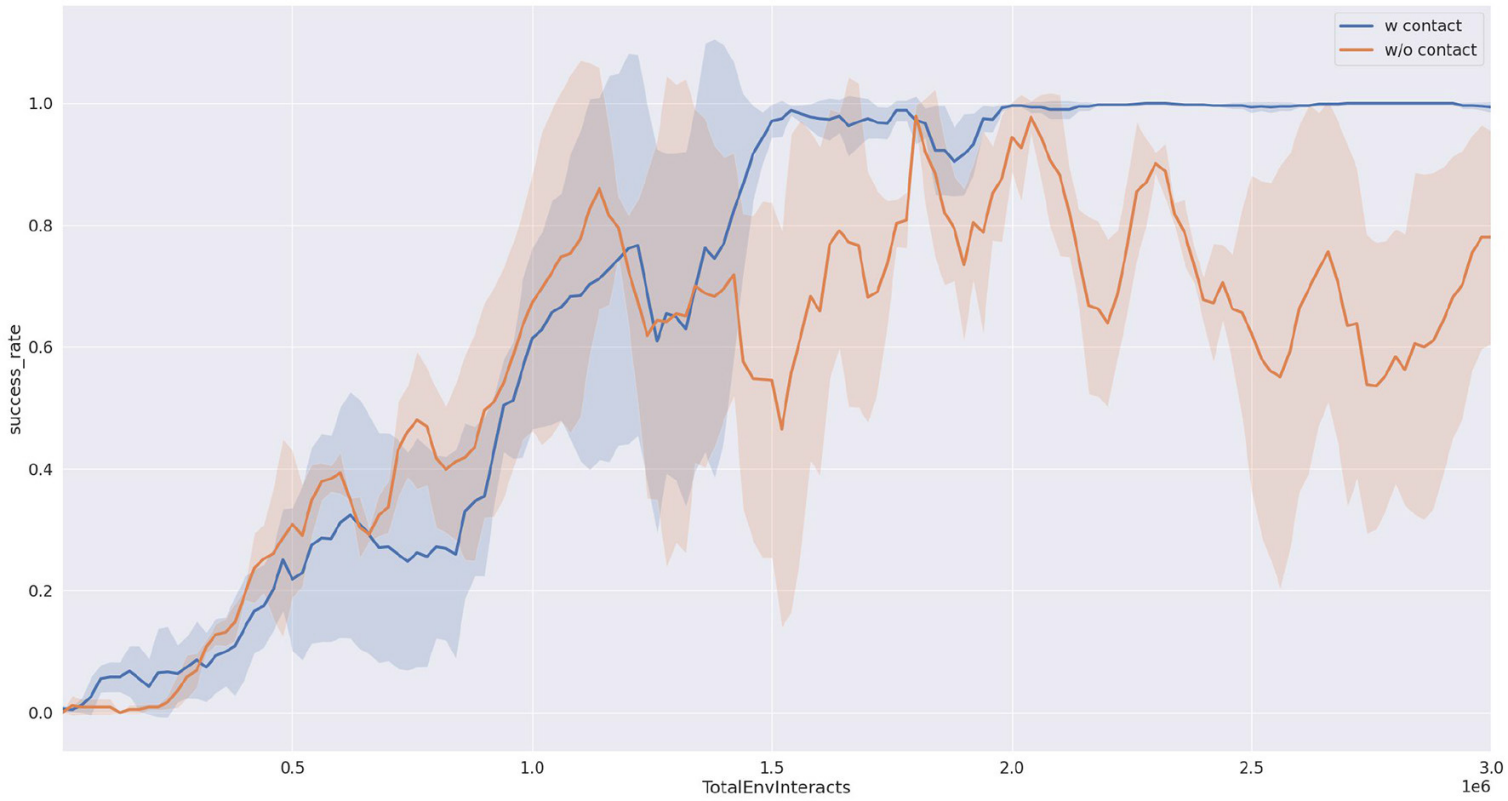
Task success rate completing 50 times tasks. The result is the average of three runs with different random seeds.

**Figure 7:**
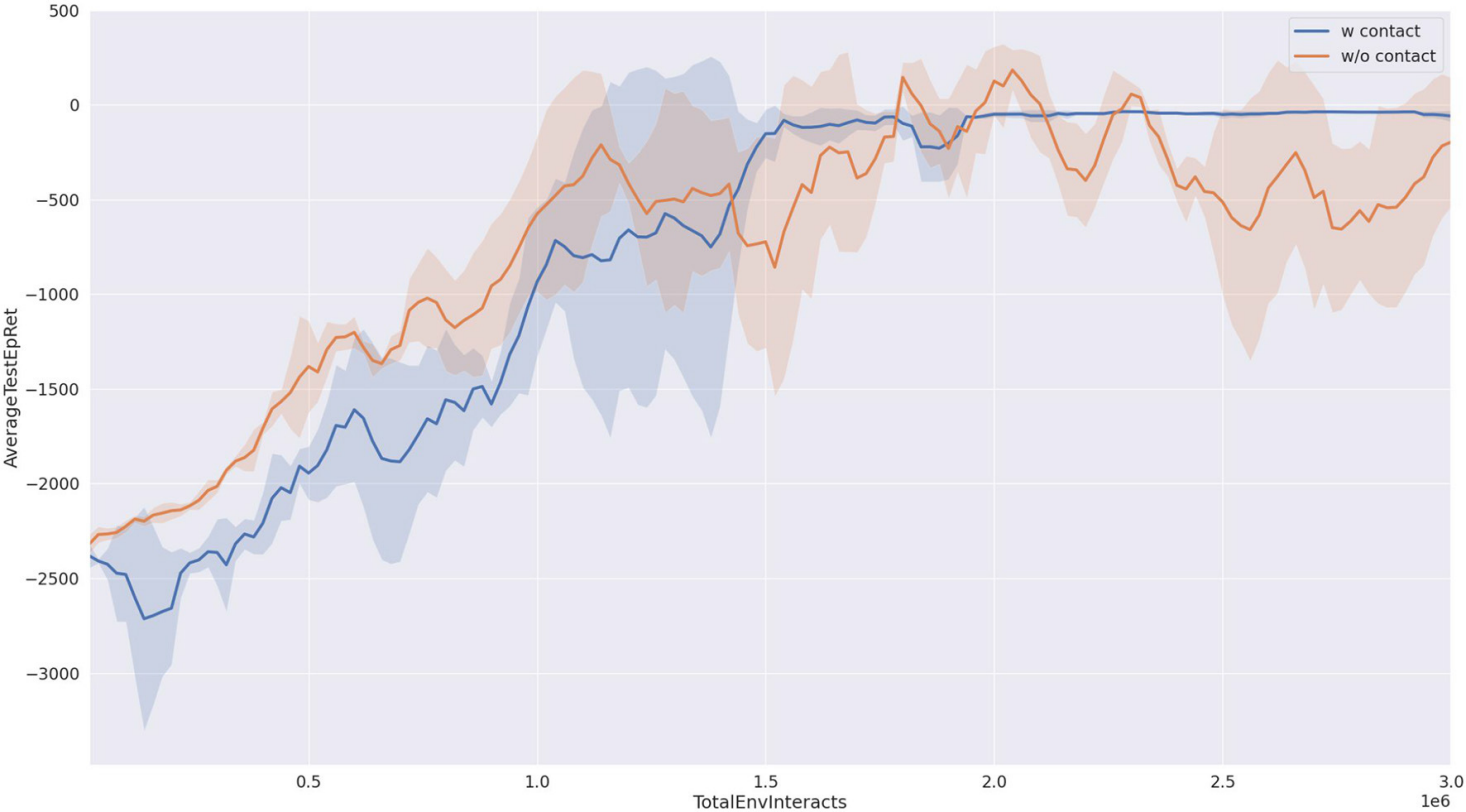
Reward curve in the training progress. The result is the average of three runs with different random seeds.

As shown in Figure 6, we compared the success rates of our proposed method and the baseline approach when pushing a block object. It can be observed from the figure that our proposed method (depicted by the blue curve) achieves a success rate approaching 100%. Furthermore, after interacting with the environment for one and a half million steps, the success rate of our proposed method stabilizes with minimal fluctuations. In contrast, the baseline method (represented by the yellow curve) exhibits success rates fluctuating around 80%, with a large variance, indicating poor strategy stability. This point also can be seen in the reward curve from Figure 7.

#### 4.3.2. Task efficiency and precision

Our proposed method not only demonstrates advantages over the baseline approach in terms of task success rate but also exhibits superiority in task efficiency and precision.

**Figure 8:**
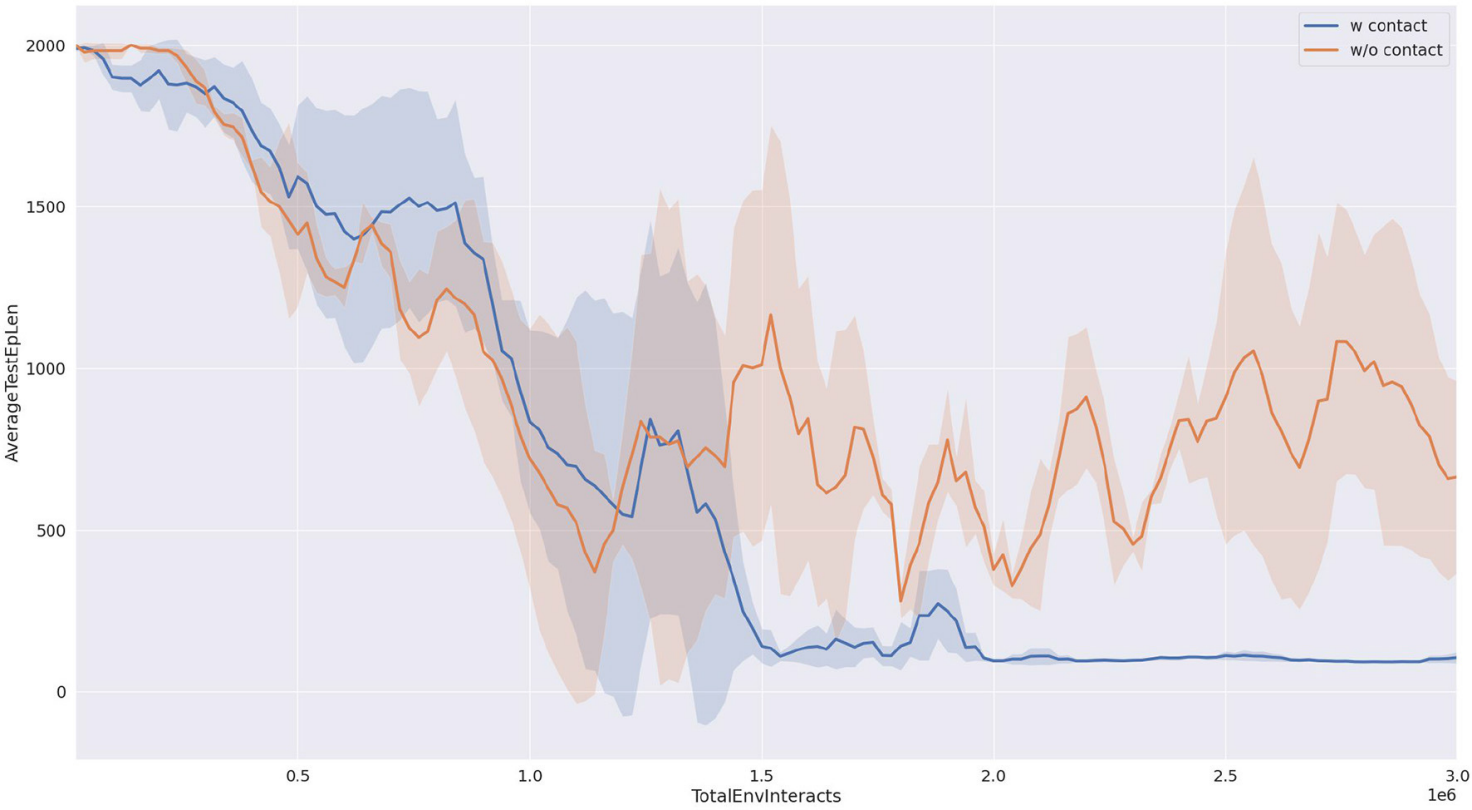
Average test length of the trajectory. The result is the average of three runs with different random seeds.

The efficiency of task execution in this study is quantified by measuring the total number of steps taken during the implementation of the learned strategy. The number of steps required to complete the pushing task is recorded during the testing phases, and the statistical results are presented in Figure 8.

**Figure 9:**
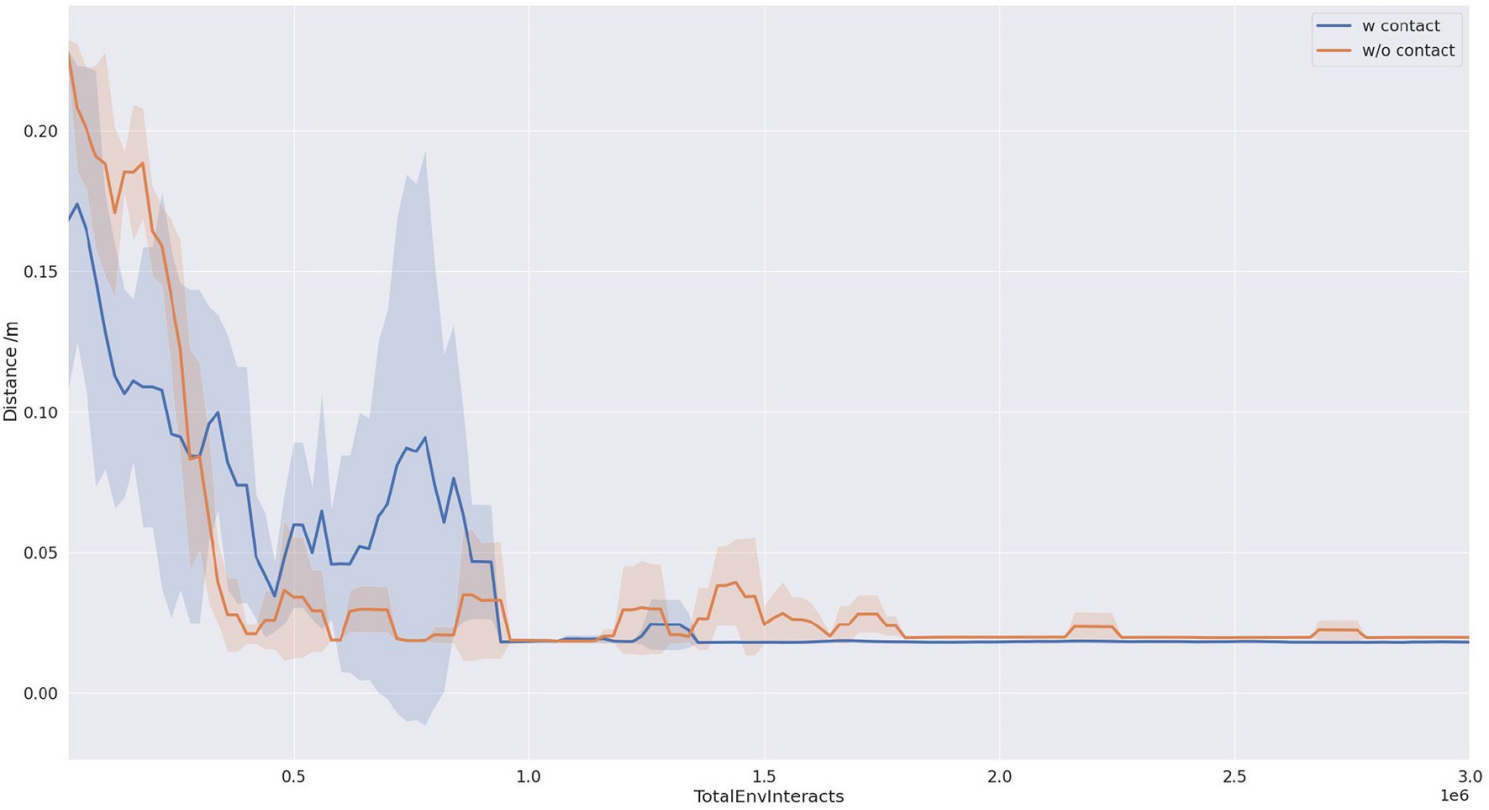
The distance from the target pose. The result is the average of three runs with different random seeds.

As shown in Figure 9, our proposed method requires significantly fewer steps to accomplish the task compared to the baseline approach, indicating higher task efficiency. This can be attributed to the reward function designed based on contact information, which encourages the strategy to interact with the object's center of mass and push it directly toward the target direction, resulting in fewer required steps. Additionally, from the graph, it can be observed that this design also contributes to an improvement in precision.

#### 4.3.3. Adaptivity performance

Although we only trained our method with a single object in the simulation, it demonstrates adaptability to multiple unseen objects. We specifically compared this aspect with the baseline method.

**Figure 10:**
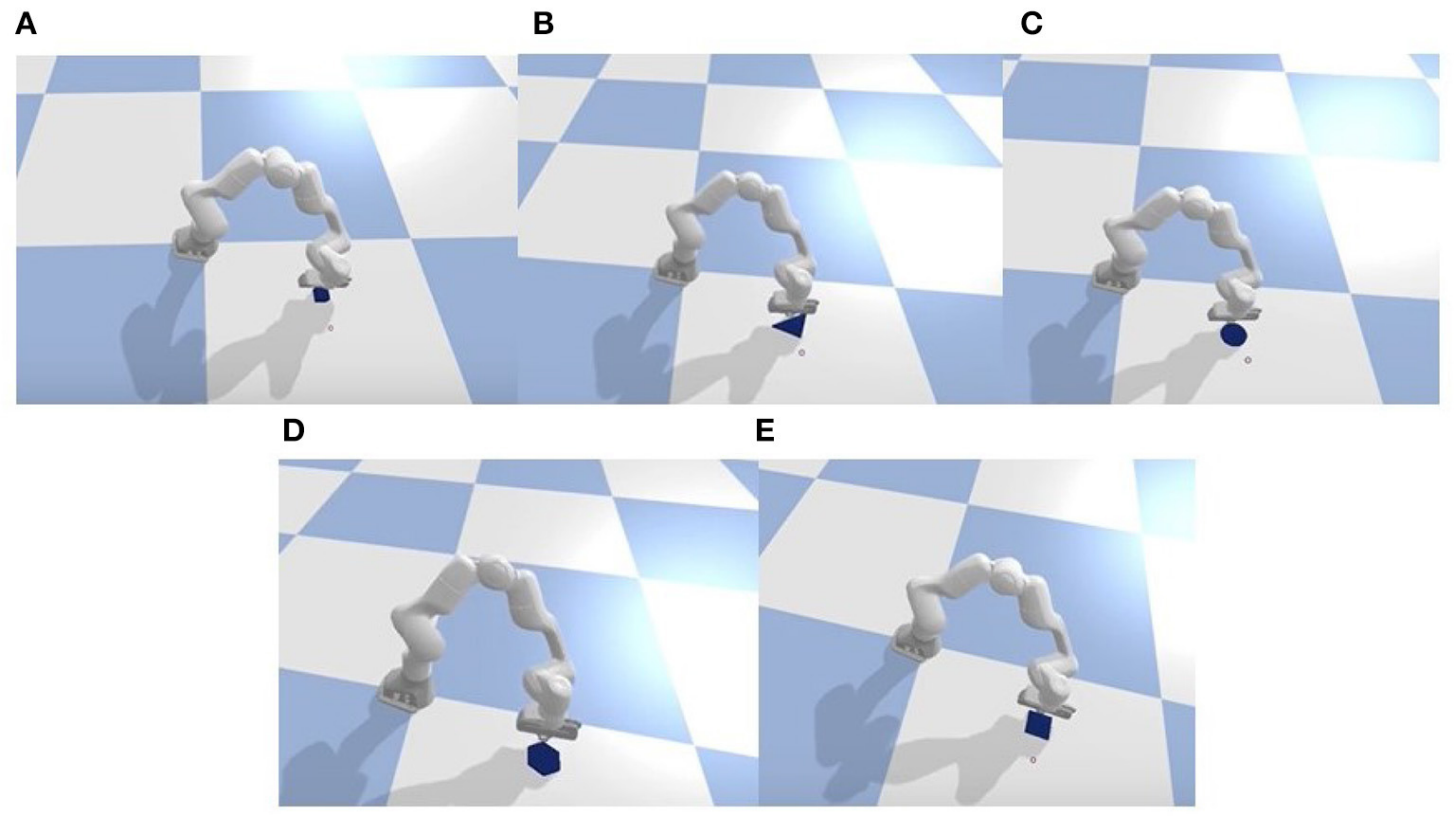
Generalization test in the simulation of four unseen objects: triangle, circle, hexagon, square. Label **(A)** is the training object and labels **(B–E)** are unseen objects.

For the adaptability experiment, we utilized four objects other than those used during training, as shown in Figure 10. Each object took 50 task trials, and the success rate was recorded. The results are presented in the table.

From the Table 2, it is evident that our proposed method exhibits excellent adaptability to previously unseen objects, showcasing robust strategy generalization. In comparison, the baseline method demonstrates relatively poorer adaptability or strategy generalization. In terms of the success rate metric, our method consistently outperforms or performs on par with the baseline method.

**Table 2:** Comparison of success rates in 50 times experiments for different objects.

**Object**	**Ours**	**Baseline**
Triangle	1.00	0.30
Circle	1.00	0.30
Hexagon	1.00	0.00
Square	1.00	0.40

#### 4.3.4. Real experiments

**Figure 11:**
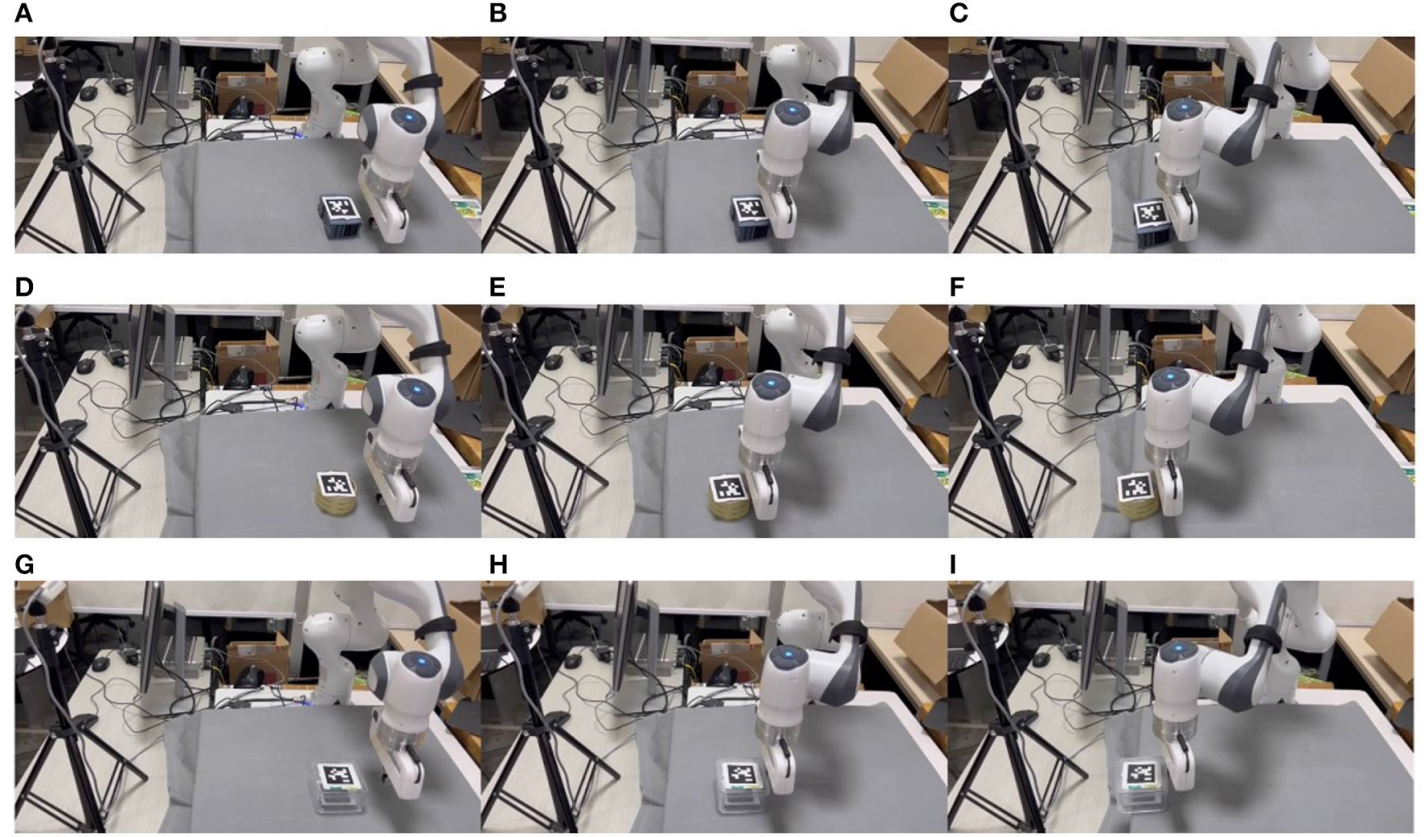
Snaps of pushing three representative objects with different mass, shape, and coefficient of friction in the real world. Labels **(A–C)** is for pushing an irregular-shaped electrical component, labels **(D–F)** is for pushing a circular tape, and labels **(G–I)** is for pushing a plastic square box.

We conducted real robot task validation for our proposed method, as illustrated in Figure 11. Our objective was to push three representative objects from random positions to the target location, which was set at the edge of the platform. This particular setup was chosen to demonstrate the role of pushing, as in our laboratory, the selected objects' dimensions exceeded the gripper's maximum width. Thus, pushing the objects to the platform's edge allowed for grasping from the thinner side.

As seen in Figure 11, our reinforcement learning strategy successfully transferred to the real-world setting, even with unseen different objects from the simulation. Nevertheless, our method accomplished the task, demonstrating its effectiveness in real-world scenarios.

**Figure 12:**
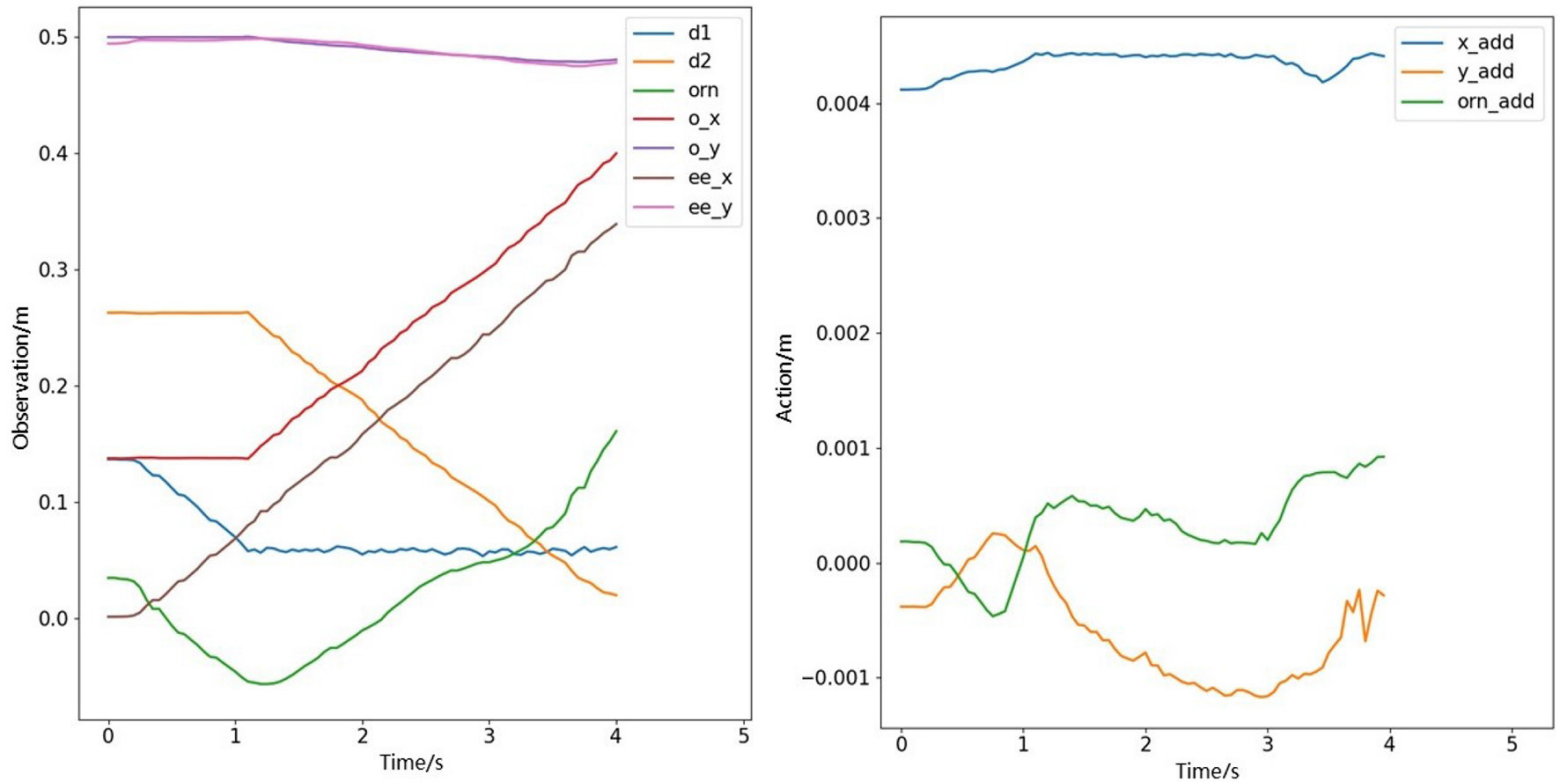
Observation and action data of pushing an object in the real world.

We further showcase the real robot's data, as depicted in Figure 12. The observation space's d2 represents the distance of the object from the target position, gradually decreasing from an initial distant position to reaching the target position. The action space outputs increments, and it can be observed that the position increment is adjusted around zero in the *y*-direction, while in the *x*-direction, the position increment maintains a constant value, allowing the robot to push the object to the target position.

## 5. Conclusion and future work

In this study, we introduced a novel Reinforcement Learning (RL)-based approach to address the task of object pushing in both simulated and real-world environments. Specifically, we utilized the Soft Actor-Critic (SAC) algorithm for training the RL policy while incorporating essential contact information between the robot and the environment through a specialized reward function. As a result, the learned policy demonstrated significantly higher task success rates in comparison to the baseline method, showcasing enhanced stability, generalization, and adaptability of the strategy. Furthermore, the successful transfer of the learned policy from simulation to real-world settings validated the efficacy of our proposed method.

In future endeavors, we aim to further enhance the policy's perceptual and decision-making capabilities by integrating additional sensory inputs, such as vision or tactile feedback.

To conclude, our study presents a promising and pragmatic approach for addressing object pushing tasks using RL, incorporating domain randomization and the SAC algorithm. The proposed method exhibits the potential for generalizing to novel objects and sets the stage for advancing research in RL-based manipulation tasks.

## Data availability statement

The original contributions presented in the study are included in the article/Supplementary material, further inquiries can be directed to the corresponding authors.

## Author contributions

SW: Methodology, Conceptualization, Data curation, Formal analysis, Investigation, Project administration, Software, Supervision, Validation, Visualization, Writing—original draft, Writing—review and editing. LS: Funding acquisition, Investigation, Resources, Supervision, Writing—review and editing. FZ: Funding acquisition, Investigation, Resources, Supervision, Visualization, Writing—review and editing. WG: Funding acquisition, Investigation, Resources, Visualization, Writing—review and editing. PW: Funding acquisition, Investigation, Resources, Visualization, Writing—review and editing.
